# The cross-talk between arterial stiffness and microvascular complications in diabetes mellitus: a systematic review of the literature

**DOI:** 10.1007/s40200-025-01647-z

**Published:** 2025-06-09

**Authors:** Styliani Partalidou, Dimitrios Patoulias, Ioannis Pantekidis, Aristeidis Kefas, Michael Doumas, Eugenia Gkaliagkousi, Manfredi Rizzo, Theodoros Dimitroulas, Panagiota Anyfanti

**Affiliations:** 1 First Department of Internal Medicine, 424 General Military Hospital of Thessaloniki, Thessaloniki, Greece; 2 2nd Propedeutic Department of Internal Medicine, Hippokration Hospital, Aristotle University of Thessaloniki, Thessaloniki, Greece; 3 Department of Trauma and Orthopaedics, Guy’s and St. Thomas’ NHS Foundation Trust, London, UK; 4 3rd Department of Internal Medicine, Papageorgiou Hospital, Aristotle University of Thessaloniki, Thessaloniki, Greece; 5 School of Medicine, Promise Department, University of Palermo, Palermo, 90100 Italy; 6 Ras Al Khaimah (RAK) Medical and Health Sciences University, Ras Al Khaimah, 11172 United Arab Emirates; 7 Fourth Department of Internal Medicine, Hippokration Hospital, Aristotle University of Thessaloniki, Thessaloniki, Greece

**Keywords:** Diabetes mellitus, Arterial stiffness, Microvascular, Pulse wave velocity, Retinopathy, Neuropathy, Albuminuria

## Abstract

Arterial stiffness (AS) is a well-established index of macrovascular damage and predicts cardiovascular complications in many diseases including diabetes mellitus (DM). DM is strongly linked with microcirculatory changes, including retinopathy, microalbuminuria and neuropathy. We aimed to review in a systematic manner the possible correlation between AS and microvascular impairment in patients with DM, type 1 or 2. We performed a systematic literature search in PubMed, Scopus and Embase database. We included studies evaluating the correlation of AS and impaired microcirculation in adult patients with type 1 or 2 DM. AS could be evaluated with pulse wave velocity, carotid-ankle vascular index, ambulatory arterial stiffness index and augmentation index. Impaired microcirculation was defined by the presence of one or more of the following: retinopathy, albuminuria, neuropathy, dermal capillary alterations. We eventually included 51 studies in our systematic review. Data were extracted by two investigators and were critically appraised with ROBINS-I tool of Cochrane Library for non-randomized trials. The majority of studies have demonstrated positive correlation between AS and microvascular impairment, usually in a dose-response association. However, most studies have focused on the association of AS with retinopathy, whereas the association with dermal microvascular alterations remains scarcely investigated. Disease duration was underlined by most authors as an independent predictor of increased AS. These findings suggest that AS should be sought upon detection of microvascular complications in patients with T1DM or T2DM; vice versa, patients with T1DM or T2DM with increased levels of AS should be screened for microvascular alterations.

## Introduction

Diabetes mellitus (DM) is a chronic, potentially disabling disease, which can cause a plethora of long-term complications associated with vascular damage in both macro- and microvasculature. Manifestations of clinically evident cardiovascular disease include coronary artery disease (CAD), cerebrovascular disease and peripheral arterial disease (PAD) [1]. On the other hand, microvasculopathy is usually present in the early stages of type 2 DM (T2DM), although in type 1 DM (T1DM) microvascular injury tends to develop later in the course of the disease [2, 3]. Microvascular complications in DM typically include three entities: proliferative or not retinopathy, microalbuminuria, and neuropathy, usually manifested as symmetrical polyneuropathy of the lower limps [4, 5].

Increased arterial stiffness (AS) constitutes structural remodeling of the large elastic vessels and has been studied diligently in the last few decades as an index of macrovascular impairment and a surrogate marker of cardiovascular disease [6]. The gold standard of AS evaluation is pulse wave velocity carotid-femoral PWV (cfPWV), although brachial-ankle PWV (baPWV), carotid-ankle vascular index (CAVI), ambulatory arterial stiffness index (AASI) and augmentation index (Aix) are also widely used as markers of AS [7–9]. Notably, the addition of PWV in models that include classical risk factors may improve cardiovascular risk prediction and enable better identification of high-risk populations [10].

**Fig. 1:**
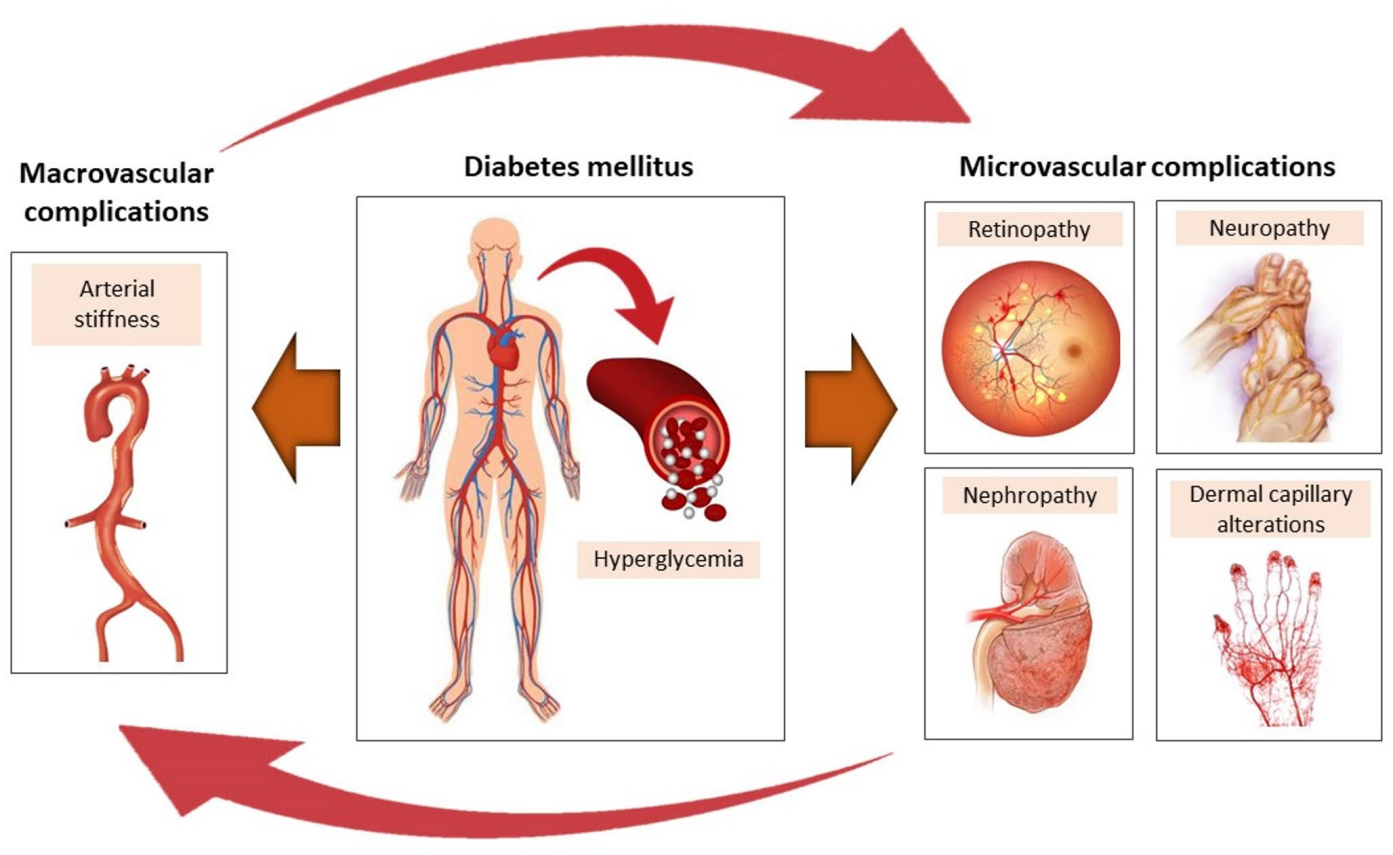
Schematic representation of the small and large artery cross-talk in patients with diabetes mellitus. High glucose concentrations induce tissue damage and generalized vascular injury through direct and indirect mechanisms that include endothelial dysfunction, oxidative stress, subclinical inflammation and accelerated atherosclerosis. While both micro- and microcirculation are simultaneously exposed to the deleterious effects of hyperglycemia, it can be hypothesized that a direct cross-talk exists between macrovascular dysfunction, i.e., large artery stiffening, and microvascular complications, i.e., retinopathy, neuropathy, nephropathy, and dermal capillary impairment, in patients with diabetes. In this vicious circle, arterial stiffness exaggerates functional and structural microvascular injury mainly through hemodynamic effects (increases in central blood pressure, pulse pressure, and flow pulsatility) to small resistance arteries. Vice versa, functional and structural microvascular abnormalities further increase total peripheral resistance and mean blood pressure that promote large artery stiffening

As a consequence of increased AS, the reflected pulse waves return from the periphery in the early systolic phase, which in turn results in reduction of dilatation and therefore an increase in systolic blood pressure, afterload, tissue oxygen needs and ischemia. Based on this hypothesis, it is reasonable to assume that increased AS may also lead to microvascular dysfunction [11]. At the same time, structural and functional alterations of the microvasculature may provoke increases in total peripheral resistance and mean blood pressure, which in turn induce large artery stiffening in the long term [12]. Notably, increased AS may denote a state of generalized vascular impairment. AS has been associated with functional and structural abnormalities in distinct microvascular beds (such as the retina, the kidney and the dermal capillary network) among patients with various diseases, such as rheumatoid arthritis [13], hypertension [14], systemic sclerosis [15] and chronic kidney disease [16, 17]. In concordance with the above, it can be hypothesized that this cross-talk between small and large arteries is evident in patients with diabetes, as displayed in Fig. 1.

Individuals suffering from DM present raised values of AS [18], and emerging evidence suggests that greater AS is associated with increased risk of developing diabetes [19]. AS has been acknowledged as a powerful independent predictor of cardiovascular disease among patients with DM [20]. Whether and to which extent the association between AS and cardiovascular complications in DM is mediated by microvascular impairment, the most prevalent form of vasculopathy in DM, remains unclarified. To this end, it is important to determine whether increased AS is associated with microvascular injury regardless of other factors in DM. Assuming that AS correlates with microvascular alterations in DM, it would be meaningful to screen for AS patients with microvascular impairment before the establishment of overt cardiovascular complications to improve cardiovascular risk stratification. Vice versa, screening for microvascular alterations might be warranted in diabetic patients with increased AS. Therefore, the aim of the current study was to review in a systematic manner the possible correlation of increased AS and microvascular complications in DM, namely retinopathy, dermal capillary alterations, microalbuminuria and/or neuropathy [21].

## Materials and methods

We conducted the current systematic review according to PRISMA guidelines [22]. The protocol for this systematic review has been registered at the Open Science Framework (https://osf.io/r4jmh).

### Literature search

We performed a literature search in PubMed, Scopus and Embase database from inception to 4th April 2024, using the following algorithm.

((((capillaroscopy[MeSH Terms]) OR (capillaroscopy)) OR ((microcirculation) OR (microcirculation[MeSH Terms]))) OR (microvascular) OR (retinopathy) OR (neuropathy) OR (microalbuminuria) OR (albuminuria)) AND ((((arterial stiffness[MeSH Terms]) OR (arterial stiffness)) OR ((vascular stiffness) OR (pulse wave velocity) OR (vascular stiffness[MeSH Terms]))) OR ((elasticity) OR (elasticity[MeSH Terms]))) AND ((diabetes mellitus) OR (diabetes mellitus[MeSH Terms])).

An English language filter was applied.

### Eligibility criteria

In the current systematic review, we included observational studies (cross-sectional, prospective, retrospective) with adult patients diagnosed with type 1 or 2 DM -irrespective of achieved glycemic control-, which evaluated the correlation of AS and impaired microcirculation, manifested as microvascular complications.

The AS could have been evaluated either with PWV (cfPWV or baPWV), CAVI, AASI or Aix. Impaired microcirculation was defined by the presence of retinopathy, microalbuminuria, neuropathy, and dermal capillary alterations. We assessed retinopathy, microalbuminuria, neuropathy as common manifestations of microvasculopathy typically encountered in the context of DM, that can be easily assessed non-invasively. More specifically, the presence of retinopathy should have been assessed by a validated ophthalmologist with fundoscopy, or optical coherence tomography (OCT)-OCT angiography. The presence of microaneurysms, infarcts, exudates, hemorrhages or neovascularization were considered as diabetic retinopathy. Microalbuminuria was defined as 30-300 mg of albumin per 24 h or 20-200mcg/min on 2 or 3 urine collections, that could not be explained by health conditions other than DM. Neuropathy should have been evaluated by history and clinical examination (absent reflexes, reduced vibration perception, nerve conduction study). Finally, studies applying videocapillaroscopy to evaluate skin capillary alterations were also eligible for the present study. Despite the fact that dermal capillary alterations have not received the same attention as markers of microvasculopathy in the context of DM in comparison to the previously described microvascular complications, emerging evidence supports the presence of a ‘‘diabetic capillaropathy’’ with promising investigational and clinical potential [23]. 

Studies that referred only to macrovascular events related to diabetes, such as peripheral vascular disease, coronary artery or cerebrovascular disease were excluded. Papers including basic research, namely animal or cellular studies were excluded. In addition, studies that referred to conditions other that DM were also excluded. These were mainly CAD, hypertension, chronic kidney disease, rheumatoid arthritis and scleroderma. Finally, we did not include studies performed in pediatric population, including adolescents with T1DM or T2DM.

### Screening process

Two blinded investigators screened the results of the algorithm for eligibility at the level of title and abstract, after duplicate removal. In case of disagreement, consensus was reached with a third senior reviewer. Then, the remaining articles were assessed at full-text level. Again, in case of disagreement, a third senior reviewer assisted.

### Data extraction and critical appraisal

Data were extracted from the studies included in the current systematic review by the two investigators in an unblinded manner. The data extracted included name of first author and year of the study, study design, total number of patients enrolled in each study, % or number of males/females, mean age, mean DM duration, type of DM, ethnicity, method of arterial stiffness evaluation, form of microvascular complication that was studied, correlation of AS and microvascular complication, any comorbidities, medication, smoking status and any other comments that the investigators had on each paper. The quality of the included studies was evaluated with the ROBINS-I Cochrane tool for non-randomized trials [24].

## Results

### Study search results

**Fig. 2:**
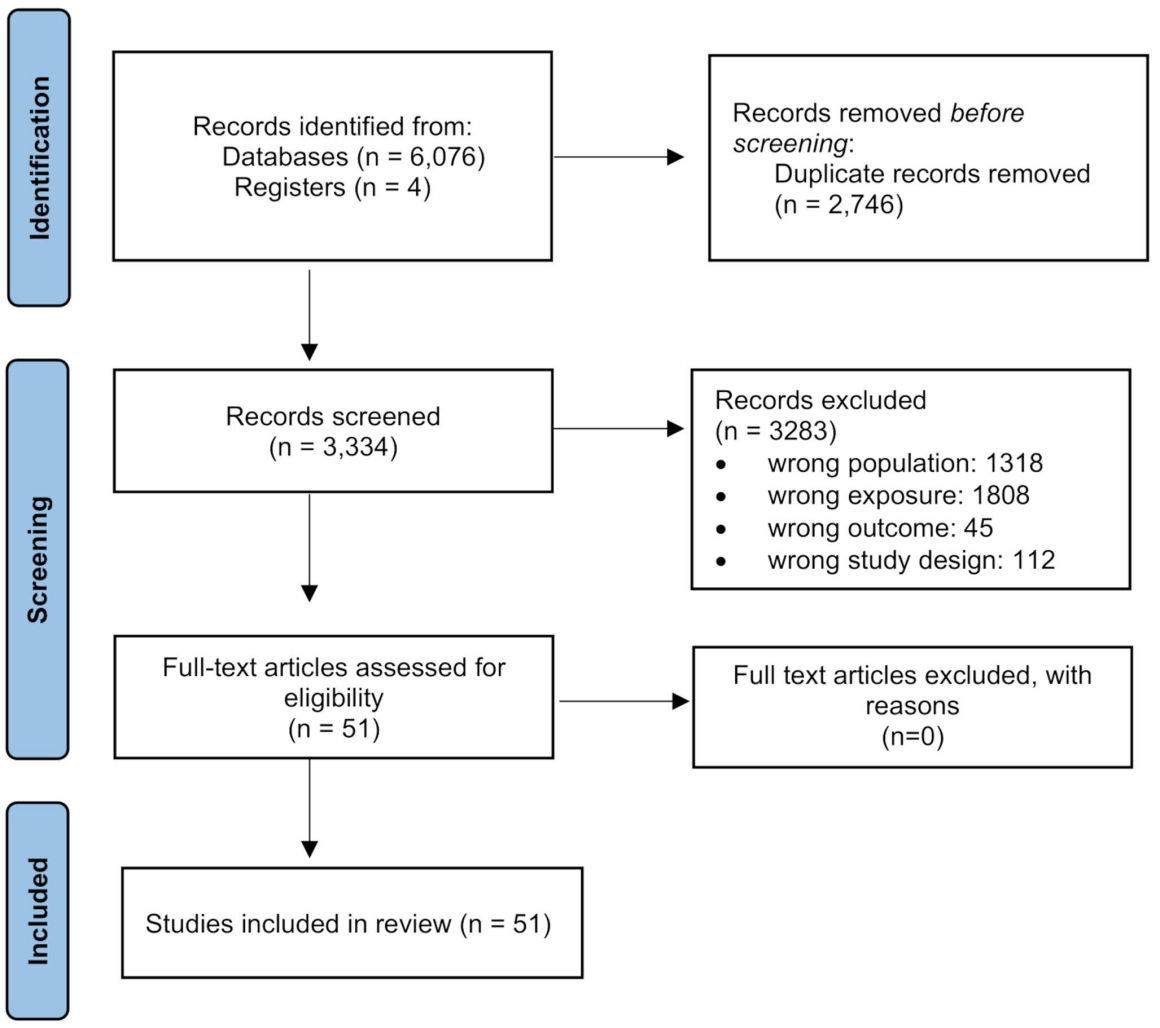
PRISMA flow diagram

The results of the algorithm implemented gave us 6,076 articles totally (1,738 articles in PubMed, 1,780 in Scopus and 2,558 in Embase). Two independent investigators proceeded in the screening and consensus was reached with a third researcher in case of disagreement. After removing the duplicates (2,746 articles), 3,330 articles remained for review at the title and abstract level and 3,283 were excluded. The rest 47 articles were evaluated as full-text and were included in the analysis. Furthermore, we screened the references from the aforementioned articles and found four additional eligible papers. Finally, 51 articles were included in our systematic review [25–75]. Figure 2 summarizes the above selection procedure in a PRISMA flowchart.

### Study characteristics

The main characteristics of the studies included in our systematic review are presented in Table 1.

**Table 1:** Study characteristics

Study	patients (total/complication)	Ethnicity	type	AS	complication	gender	mean duration	mean age	comorbidities	smoking	outcome
An, 2021	2473/734	Chinese	2	baPWV	ret	NR	NR	NR	NR	NR	positive OR = 1.59
Anan, 2007	36/NR	Japan	2	baPWV	alb	22 ♂	9.6y	56	Hypertension (67%), dyslipidemia (44%)	39%	positive *r* = 0.53
Ando, 2017	166/NR	Japan	2	CAVI	neuro	64.5% ♂	13y	59	Hypertension (48%), dyslipidemia (74%)	59%	positive
Ari Widjaja, 2024	170/NR	Indonesia	2	baPWV	ret	42.9% ♂	6.21y	54.43	Hypertension (41.13%)	0	positive
Aso, 2003	90/NR	Japan	2	baPWV	all	43 ♂	13.7y	60.3	Hypertension (48%), dyslipidemia (54%)	33%	positive OR = 20.9(ret), OR = 7.6(alb), OR = 15.2(neuro)
Avci, 2014	161/69	Turkey	2	PWV	neuro	81 ♂	60 m (neuro)/36m (no neuro)	55(neuro)/51 (no neuro)	Hypertension (26 neuro/35 no neuro)	21%(neuro)/23%(no neuro)	positive
Bouchi, 2011	461/122	Japan	2	cfPWV	alb	262 ♂	12y	59	CVD, Hypertension, dyslipidemia	24%	positive HR = 1.23
Cardoso, 2009	482/26	NR	2	PWV	Ret & neuro	62% ♂	13y (increased PWV)/6y others	67(increased PWV)/59 others	Hypertension, dyslipidemia, CVD, cerebrovascular disease	NR	positive ret(OR = 3.83), neuro(OR = 1.79)
Chen, 2015	447/53%neuro-40% neuro & Hypertension	France	2	cfPWV	neuro	NR	NR	NR	Hypertension	NR	positive
Choi, 2010	673/318	Korea	2	baPWV	alb	215 ♂	8.9y	68.2	Hypertension (487)	106	positive
Drinkwater, 2021	524/164 (eyes)	Australia	2	cfPWV	ret	153 ♂	15y	72	NR	NR	positive OR = 1.13
Eveilleaue, 2023	83/61	NR	1	PWV	Ret	59% ♂	26y	42	NR	NR	positive
Gordin, 2012	622/67	nationawide	1	PWV & AI	Ret & alb	NR	31y	40.6	Hypertension, CVD	NR	Positive OR = 1.04(ret) & OR = 1.06(alb)
Ha, 2012	692/NR	NR	2	baPWV	neuro	48.5% (low PWV)/ 38% high PWV ♂	7.3y in low PWV/8.5y in high PWV	51.5 low PWV/62 high PWV	Hypertension, dyslipidemia	26.3% in low PWV/23.5% in high PWV	positive with OR = 1.61 (PWV > 1600)
Hayfron-Benjamin, 2021	986/NR	nationwide	2	PWV	alb	505 ♂	8.21y	55.58	Hypertension	230	positive OR = 2.55
Huang, 2014	578/NR	Chinese	2	baPWV	alb	339 ♂	5y	58.4	Hypertension, dyslipidemia	NR	positive OR-1.317 & *r* = 0.137
Kim, 2011	320/64 increased CAVI	Korea	2	CAVI	all	53% ♂	7y (increased CAVI)/3 others	61 (increased CAVI)/58 others	Hypertension, dyslipidemia	7.6%	Positive OR = 2.47(alb), OR = 2.03(neuro) No correlation for ret
Kim, 2019	424/93	Korea	2	baPWV	alb	173 (no alb)/57 alb ♂	7.5y (no alb)/10.4 (alb)	58.2 (no alb)/ 59.5 (alb)	Hypertension, dyslipidemia	14.2% (no alb)/26.9% (alb)	Positive *r* = 0.79 & OR = 10.89
Lamacchia, 2019	299/74	Caucasians	2	CAVI	ret	166 ♂	11.9y	60.4	Hypertension, dyslipidemia	NR	positive
Laugesen, 2009	102/34	Denmark	1	AASI	alb	24 ♂	NR	30	Hypertension	42	positive
Lim, 2014	95/NR	Chinese	2	AI	ret	77% ♂	15.5y	60	Hypertension (114)	25 current/35 former	positive
Liu, 2020	846/214	Taiwan	2	baPWV	ret	154(NDR)/33(NPRR)/13(PDR) ♂	11.3(NDR)/13.7(NPDR)/17.3(PDR) y	65.3 (NDR)/68(NPDR)/63.8(PDR)	Hypertension, dyslipidemia	7.3%(NDR)/6.2%(NPDR)/8.9%(PDR)	Positive OR = 6.15(PDR)
Ogawa, 2005	1004/240	Japan	2	baPWV	ret	148 (ret)/531(no ret) ♂	14(ret)/8(no ret) y	61.3(ret)/60 (no ret)	Hypertension	NR	positive
Pei, 2023	322/154(ret)-131(alb)-137(neuro)	Chinese	2	baPWV	all	192 ♂	6.3y	56.7	Hypertension	114	positive
Prince, 2010	130/25	Pittsburgh	1	PWV, Aix	alb	46.2%(no alb)/60% (alb) ♂	36.1y	44.3	NR	NR	No correlation
Rema, 2004	590/116	Asian-Indian	2	Aix	ret	199 (no ret)/58(ret)♂	3 (no ret)/7 (ret)y	53	Hypertension, CVD, alb	43 (no ret)/ 8 (ret)	positive
Shin, 2013	218/104	Korea	2	PWV	alb	53% ♂	6 m	56	Dyslipidemia	18 (alb)/ 56 (no alb)	positive with *r* = 0.47
Siasos, 2015	300/92	Greek	2	cfPWV, Aix	ret	NR	NR	NR	NR	NR	positive
Smith, 2005	134/NR	nationwide	2	PWV	alb	89 ♂	9y	60.6	CVD (12), cerebrovascular (11), both (8), dyslipidemia, hypertension	22	positive with *r* = 0.235
Solanki, 2021	47/33	India	2	PWV	ret	28 ♂	8.83y	61.2	Hypertension	0	No association
Suh, 2010	100/20	Korea	2 (98%) & 1 (2%)	baPWV	neuro	51 ♂	6.3y	55.4	Hypertension	NR	positive
Szczybra, 2015	42/NR	Poland	1	cfPWV	neuro	17♂	20.6y	37.1	Hypertension	NR	positive
Tanaka, 2018	292/185	Japan	2	baPWV	neuro	100% ♂	9.9y	60.6	Hypertension, dyslipidemia	NR	positive
Tanokuchi, 1995	107/NR	NR	2	PWV	ret, alb	58 ♂	10.96y	59.11	Hypertension	47	No association
Tapp, 2018	53,094/NR	British	2	ASI	ret	NR	NR	55	NR	NR	positive
Tentolouris, 2017	381/107	Greek	2	cfPWV	neuro	NR	NR	NR	NR	NR	Positive with OR = 1.174
Teoh, 2011	860/141(neur0)-267(ret)-188(alb)	Scotish	2	PWV	all	NR	NR	69	NR	NR	positive
Theilade, 2012	635/152	Caucasians	1	cfPWV	all	349 ♂	32y	54	Hypertension	19%	positive
Tjessem, 2017	151/NR	NR	1	AASI	alb, ret	100% women	13y	28	NR	NR	Positive but lost post-partum
Tougaard, 2020	633/NR	NR	1	cfPWV	alb	55% ♂	32y	54	Hypertension	NR	Positive with HR = 1.59
Turan, 2019	180/82	Turkey	2	AASI	alb	68 ♂	11.6y	58.5	Hypertension	NR	positive with OR = 4.19
van der Heide, 2021	2434/NR	Netherlands	2	cfPWV	ret	51.4% ♂	NR	59.8	CVD, dyslipidemia, hypertension	315 current/1302 former	No association
Van Sloten, 2014	524/NR	France/Netherlands	2	cfPWV	skin	45.2% ♂	7y	61	CVD, dyslipidemia, hypertension	8.8% current/15.8% current	No association
Van Sloten, 2014	737/NR	Netherlands	2	cfPWV	skin	54.8% ♂	NR	59.7	NR	NR	No association
Yeboah, 2018	240/47	Africans	2	PWV & CAVI	neuro	175 ♂	10.4y	54.1	Hypertension	NR	Positive only for CAVI
Yokoyama, 2003	346/119	Japan	2	baPWV	alb	61% ♂	NR	61	CVD, dyslipidemia, Hypertension	37%	Positive with R^2^ = 0.42
Yokoyama, 2004	306/106	Japan	2	baPWV	alb	64% ♂	8y	58	CVD, dyslipidemia, Hypertension	44%	positive
Yokoyama, 2007	294/93	Japan	2	baPWV	neuro	72% ♂	9y	59	AH, dyslipidemia, ret, nephropathy	29%	positive
Yun, 2011	605/114	Korea	2	baPWV	ret	144(no ret)/39(ret) ♂	7.7(no ret)/12.9(ret)y	68.7(no ret)/64.4(ret)	Hypertension, dyslipidemia	NR	positive
Zhang, 2019	1203/271 NPDR &108 PDR	Asians	2	cfPWV & Aix	Ret	623 ♂	14.7y	59.3	Hypertension, neuropathy	325	positive with OR = 1.11(NPDR) & OR = 1.15(PDR)
Zhang, 2021	120/60	Chinese	2	baPWV	alb	26 ♂	10y	61.4	NR	NR	positive

AASI = ambulatory arterial stiffness index, Aix = augmentation index, alb = microalbuminuria, AS = arterial stiffness, CAVI = carotid-ankle vascular index, CVD = cardiovascular disease, HR = hazard ratio, NR: not reported, OR = odds ratio, neuro = diabetic neuropathy, ret = diabetic retinopathy, PDR = proliferative diabetic retinopathy, baPWV = branchial-ankle pulse wave velocity, cfPWV = carotid-femoral pulse wave velocity PWV = pulse wave velocity, NPDR = non-proliferative diabetic retinopathy, r/R^2^ = correlation index. “All” refers to the concomitant evalution of neuropathy, retinopathy and nephropathy microvascular alterations

Thirty-seven studies were cross-sectional, nine studies included a control group, three were prospective and two studies were retrospective. Regarding the ethnicity, there was multi-national representation, with Caucasians, Asians and Africans as well. Forty-two studies presented data of patients with T2DM, 8 studies involved patients with T1DM, while one study had both, with the majority, though (98%), being T2DM. Finally, one of the studies with T1DM included women during pregnancy.

AS was evaluated mostly via cfPWV or baPWV (44 studies) and the rest used CAVI, AASI or Aix. Three studies examined the AS using combined methods.

### Risk of bias assessment

**Fig. 3:**
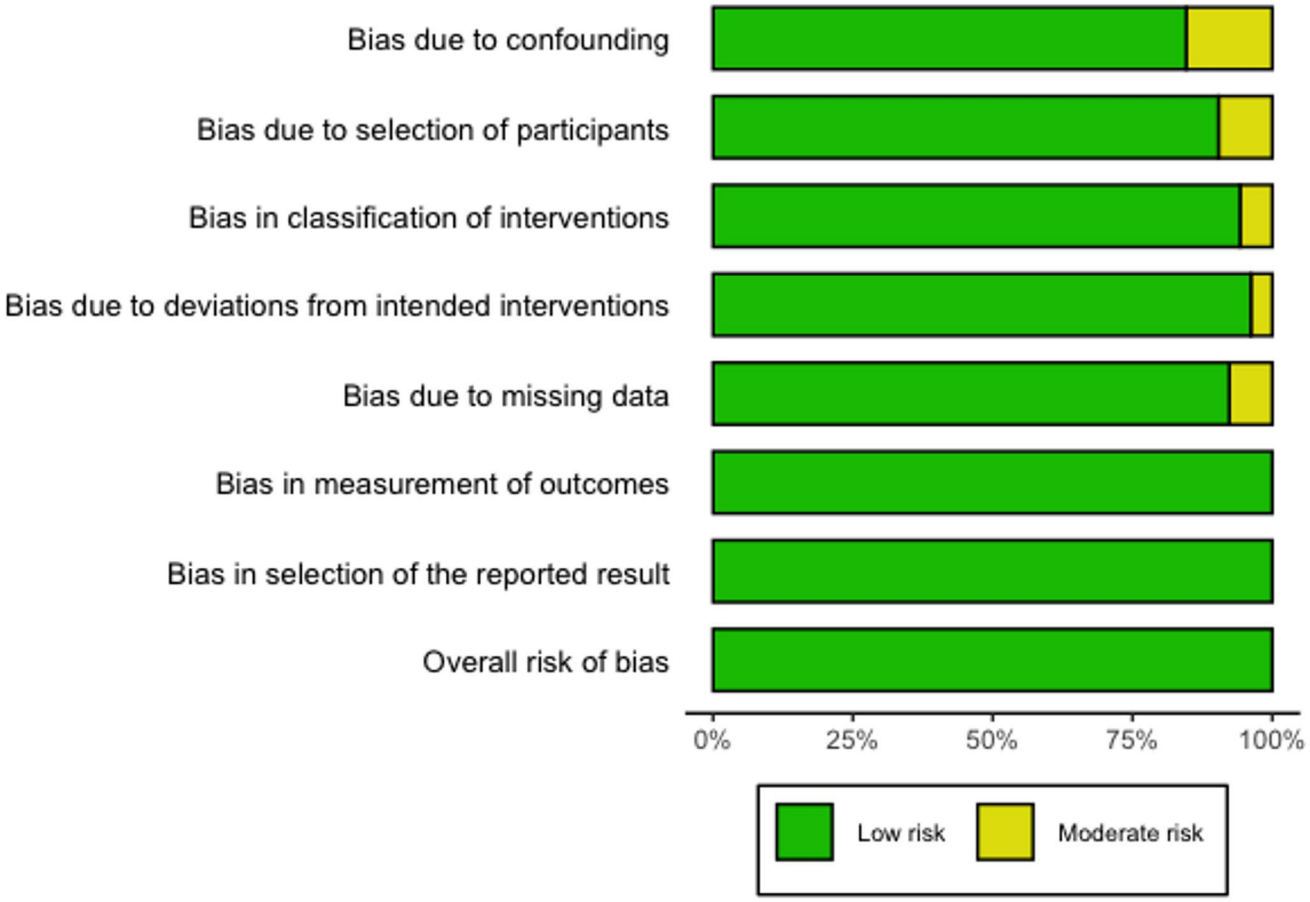
Risk of bias assessment

The investigators who extracted the data, also assessed the risk of bias of each study independently, using the ROBINS-I tool of Cochrane Library for non-randomized trials; evaluating seven domains: bias due to confounding, bias in selection of participants, bias in classification of interventions, bias due to deviations from intended interventions, bias due to missing data, bias in measurement of outcomes, and bias in selection of the reported result. An overall risk of bias judgment - “low,” “moderate,” “serious,” or “critical” - was based per the ROBINS-I guidance. The Cohen’s kappa coefficient (κ = 0.78) indicated substantial inter-reviewer agreement. Discrepancies were resolved through discussion between the two assessors, and when consensus could not be reached, a third reviewer adjudicated the final decision. Consensus was reached with the assistance of a third investigator. A bar plot of this assessment is presented in Fig. 3. No study was excluded due to high risk of bias.

#### Diabetic retinopathy

Twenty-three studies examined the presence of retinopathy, and many also proceeded in detecting whether there was a dose-response association of the AS with the degree of retinopathy (proliferative or not) [26, 27, 33, 35, 39, 41, 43, 45, 46, 49, 50, 53, 55, 56, 64, 65, 67, 68, 70, 72–75]. The results were expressed as odds ratio (OR), hazard ratio (HR), correlation index (r or R²), while in some studies it was just examined whether the difference in the group of patients presenting the complication was statistically significant compared to the group of diabetic patients not experiencing the particular complication. Out of 23 studies, 19 presented a statistically significant association between AS and the presence of retinopathy, and 11 of these reported that as arterial stiffness was higher, the retinopathy tended to be more severe. Four of these studies involved patients with T1DM [33, 56, 68, 70]. However, Tjessem et al., who studied pregnant patients with known T1DM, note that this association was lost postpartum [70]. Finally, four studies found no correlation between AS and the presence of retinopathy [35, 39, 53, 73].

It is worth noting that five of the studies that reported a positive correlation between AS and diabetic retinopathy also underlined that the DM duration was significantly higher in this population [26, 27, 33, 45, 72, 73]. In addition, two studies point out that poor glycemic control with high levels of hemoglobin A1c (HbA1c) is also associated with higher AS and, consequently, diabetic retinopathy [26, 72]. In line with these, the studies of Liu and Cardoso et al. report that patients presenting increased AS and diabetic retinopathy also appear to have higher blood pressure, mainly systolic blood pressure [45, 72]. Finally, Ogawa et al. is the only study that associates female sex with higher odds for diabetic retinopathy [55].

#### Microalbuminuria

Twenty-three studies examined the presence of microalbuminuria and its correlation with AS [25, 29, 31–33, 35, 40, 42, 44, 47, 48, 50–52, 56, 57, 59, 61, 63, 70, 71, 73, 75]. Six of them included patients with T1DM [32, 33, 44, 48, 56, 70]. The results, again, were expressed as OR, HR, correlation index (r), mean difference, or evaluation of statistical significance between diabetic patients with albuminuria and diabetic patients without albuminuria. Patients being transplanted or under dialysis were excluded from all studies, and cases of renal impairment due to non-DM relative causes were also excluded. Out of the 23 studies, 20 found a statistically significant correlation between AS and the presence of microalbuminuria in patients with DM. Furthermore, two studies underlined that the degree of albuminuria was more severe as the AS was greater [48, 70]. However, in one of these two studies, Tjessem et al. evaluated patients with T1DM during pregnancy and pointed out this association did not remain significant postpartum [70]. Notably, only Tanokuchi et al. found no correlation between AS and the presence of microalbuminuria [35].

Again, as in diabetic retinopathy, many studies recognize DM duration as an independent factor for microalbuminuria development and progression [25, 31, 32, 40, 52, 59, 73]. Shin et al. included Korean patients newly diagnosed with T2DM (mean disease duration six months) and also found a positive correlation between AS and the presence of microalbuminuria [42]. Beyond disease duration, many studies have reported significantly raised AS and microalbuminuria with advanced age [25, 29, 31, 32, 52]. Higher systolic blood pressure was independently associated with higher AS in three studies [29, 32, 59]. Finally, only one study found a positive correlation of higher cfPWV with female gender [59].

#### Neuropathy

Sixteen studies examined the presence of diabetic neuropathy and its potential correlation with increased AS [28, 30, 33, 34, 36–38, 50, 54, 58, 60–62, 72, 73, 75]. Only two of these studies involved patients with T1DM. Theilade et al. used abnormal heart rate variability as an index of autonomic nervous system disease, and found a significant association with PWV in T1DM [33]. Szczyrba et al. found higher PWV in T1DM patients with diabetic symmetric polyneuropathy compared to those without [37]. In studies recruiting T2DM patients, the evaluation of diabetic neuropathy was conducted with variable measures. For instance, Tanaka et al. performed nerve conduction velocity to assess median and tibial nerves, as well as median and sural nerves as sensory nerve [36]. Avci et al. assessed presence of peripheral neuropathy based on clinical examination by a neurologist [60], and Cardoso et al. applied clinical tests of cardiovascular dysautonomy [72]. Aso et al. evaluated diabetic peripheral neuropathy with the use of Semmes-Weinstein monofilaments [61]. The association between AS and neuropathy was expressed with OR, correlation index (r), or comparison between patients with neuropathy and patients without this particular complication. It is noteworthy that no study reported an absence of correlation between AS and neuropathy. However, Yeboah et al. reported that the association with large-fibre nerve function, as assessed by vibration perception threshold using a neurothesiometer, was only present when AS was evaluated with CAVI and not with PWV [30].

Furthermore, unlike retinopathy and microalbuminuria, there was no report of a dose-response association. Four studies found that AS tended to be more increased in older patients with neuropathy [28, 34, 54, 58]. Additionally, in line with the other two microvascular complications (microalbuminuria and retinopathy), DM duration and poor glycemic control with high levels of HbA1c were found to be independent factors of increased AS and, consequently, progression to neuropathy [28, 34, 54, 58, 60, 73, 75]. High blood pressure was also associated with advanced AS in four out of 15 studies [37, 54, 58, 73]. Finally, gender association appears to be controversial, as Ha et al. underlined female gender to be a risk factor for neuropathy development, in contrast to the study of Tentolouris et al., who found male sex to be significantly associated with increased AS [34, 54].

#### Skin microcirculation

Finally, two population-based cohort studies were included in this systematic review that explored the correlation between AS and skin microcirculation. Both studies emerge from the same study group (Van Sloten et al.) and incorporate essentially the same population. The first study incorporated data from two studies: SUVIMAX2 study (*n* = 284), which involved non-diabetic patients with arterial hypertension or CAD, and Maastricht study (*n* = 737), which included a minority of patients with T2DM (28.8%). AS was determined by cfPWV, and skin capillaroscopy was applied to determine capillary density at rest and during reactive hyperemia and venous congestion. Laser Doppler flowmetry was additionally applied to assess acetylcholine- and local heating-induced vasoreactivity, and skin flowmotion. In the total population, no association was detected between AS and skin microcirculation after adjustment for age, sex, mean arterial pressure, heart rate and cardiovascular risk both at rest and after stimulation. Results were qualitatively similar in individuals with T2DM. The second study exploring the association between AS and nailfold capillaroscopic parameters was the Maastricht study analyzed separately and alone. Similarly, no association was found, after adjustment for multiple cardiovascular risk factors, between skin microcirculation and AS, irrespective of the presence of DM [66, 69].

#### Multiple microvascular impairment

Of the included studies, eight have reported on more than one microvascular complication in association with AS [33, 35, 50, 56, 61, 70, 72, 73]. One study reported on the association with retinopathy plus neuropathy [72], three on the association with retinopathy plus nephropathy [35, 56, 70], and the remaining four on the association with retinopathy, neuropathy plus albuminuria [33, 50, 61, 73]. Two studies included patients with T1DM [33, 56], one study included pregnant women with T1DM [70], and the rest patients with T2DM. With the only exception of the oldest study by Tanokuchi et al. [35], the association between AS and concomitant microvascular injury was confirmed in more than one microvascular beds.

## Discussion

In the current study we explored the potential correlation between AS and the most common microvascular complications of DM, namely retinopathy, neuropathy and microalbuminuria, in a systematical manner. Even though diabetic skin microcirculation changes are not included amongst the typical microvascular complications related to diabetes, we included a small number of relevant studies mainly due to the emerging role of nailfold videocapillaroscopy in the assessment of peripheral microangiopathy. The results of our analysis demonstrated a positive correlation between AS and the presence of one or more microvascular complications (retinopathy, neuropathy, microalbuminuria) in patients with DM, type 1 or 2. In multivariate analysis, most studies found that the DM duration had a linear association with the AS and, consequently the microvascular complication(s) studied. However, there are some further significant points to be considered.

First, most of the studies have focused on the association between AS and retinopathy, indicating that higher grade of AS is associated with worse degree of retinopathy, especially proliferative retinopathy at highest values of AS. Fewer studies report on the association of AS with microalbuminuria and far fewer with neuropathy, although both represent common microvascular complications encountered early in the course of the disease. Importantly, the association between AS and skin microcirculatory disorders remains essentially understudied and is only vaguely understood, and no safe conclusions can be extracted from available studies.

Second, the vast majority of the studies have focused on T2DM rather than T1DM. Individuals with T1DM and T2DM share several physiological dysfunctions driven by hyperglycemia, such as endothelial dysfunction, low-grade inflammation, oxidative stress, and metabolic inflexibility, eventually leading to mutual cardiometabolic and vascular complications [4, 41]. Nevertheless, it could be hypothesized that the autoimmune nature of T1DM may modify the association between large artery dysfunction and microvascular alterations, and hence, the association between AS and microvascular complications in T1DM warrants further investigation.

Third, the included studies have exclusively reported on patients with long-term DM, with the only exception of Shin et al., who examined newly diagnosed patients with T2DM and confirmed a positive correlation of increased PWV with the presence of microalbuminuria [42]. This finding indicates that clinicians should seek increased AS and the presence of microvascular complications in patients with T2DM early in the disease course. However, this is a single study and warrants further verification for this and other forms of microvascular alterations, both for patients with T2DM and T1DM. Hence, it remains unclear whether the association between AS and other microvascular complications is observed from the early stages of the disease, pending confirmation from further studies.

Previous studies support that women with DM tend to present worse cardiovascular profiles and progress to cardiovascular complications more rapidly compared to men, after the diagnosis of DM. In fact, there is evidence that women have more increased BMI and poor control of lipid and blood pressure control than men [76–79]. In line with these, three of the included studies in this systematic review found that female gender was an independent risk factor for increased AS and the development of diabetic microvascular complications. Specifically, Bouchi et al. examined the correlation of increased cfPWV and the progression of albuminuria (from normal urinary albumin excretion to microalbuminuria and then from microalbuminuria to macroalbuminuria) in Japanese patients with T2DM and concluded that women presented greater HR [59]. Next, Ha et al. found that women with T2DM and increased baPWV had more odds of suffering from neuropathy compared to men [54]. Finally, Ogawa et al., who studied the correlation of AS and diabetic retinopathy, also underlined that diabetic Japanese women with increased baPWV were more likely to present retinopathy in comparison with men [55]. It is apparent that all diabetic microvascular complications may be more common in women, implying that a more diligent effort might be placed in this subgroup of diabetic patients to detect these adverse complications and provide appropriate management.

Notably, studies that reported no correlation of AS with some microvascular impairments may be subject to methodological limitations that could at least partially account for the non-significant findings. For instance, Kim et al. found a positive association with increased CAVI and neuropathy as well as microalbuminuria, whereas retinopathy was the only complication that did not correlate with advanced AS. The method of evaluating retinopathy in this study was fundoscopy, which is a highly subjective approach depending on the expertise of the ophthalmologist conducting it [73]. Other limitations may be related to the inherent characteristics of the study populations. Solanki et al. excluded smokers and patients under insulin treatment, which could be considered a possible selection bias. In fact, patients using insulin injections may be those with poor glycemic control, which is one of the risk factors for presenting increased AS, as shown in plenty of the studies included [39]. Similarly, Tanokuchi et al. involved only non-insulin-dependent diabetic patients [35].

Indeed, improved glycemic control, along with reductions in blood pressure and heart rate, has been identified as the most important contributor to attenuation or prevention of AS progression in patients with T2DM, as shown in the Rio de Janeiro T2DM cohort study [80]. In the present systematic review, many of the included studies have pointed out the usual coexistence of increased AS and high blood pressure, usually the systolic component, independently of other comorbidities and anthropometric characteristics (e.g., BMI and waist circumference). Hypertension is a major driver of mutual micro- and macrovascular injury [81], and as such, appropriate hypertension control in DM emerges as extremely important to attenuate or even reverse AS and related microvascular complications.

Most studies indicated a linear association between AS values and the degree of microvascular complications. However, a cut-off value for AS, above which the possibility of underlying microvascular impairment is high, might be of clinical utility. Interestingly, two studies proposed a cut-off value for baPWV that could predict the development of the microvascular complication under investigation. More specifically, Kim et al. suggested that the appropriate value for baPWV to predict microalbuminuria (20–200 micrograms/min) was 1700 cm/sec [51] Similarly, Liu et al. proposed the value of 18.58 m/sec as the cut-off to detect retinopathy in patients with DM [45]. Apparently, the two values are quite similar, despite referring to different microvascular complications. Further studies are needed to set firm cut-off values for AS among patients with DM that indicate a state of generalized macro- and microvascular impairment.

While our study offers some new insights on the associations between AS and microvascular complications, it also points towards future research directions. Most of the studies were conducted before the introduction of newer antidiabetic treatments [e.g., sodium-glucose cotransporter-2 inhibitors (SGLT2i), glucagon-like peptide-1 receptor agonists (GLP-1RAs)]. These agents have revolutionized the field of antidiabetic management, as they offer a unique route of cardio- and nephro-protection [82, 83]. It can be therefore reasonably hypothesized that their use would mitigate the association between AS and microvascular complications in patients with DM, potentially mediating their beneficial effects in terms of cardiovascular health. However, this hypothesis warrants validation in future, appropriately designed studies.

Strengths of the present systematic review include the incorporation of a large number of studies, both older and more recent, that involved a great number of patients from all over the world. All major microvascular complications of DM were reviewed in association with AS. Considering its emerging role in the field of microvascular assessment, impairment of the dermal capillary network was reviewed for the first time in association with AS in DM. Studies reporting on either T1DM or T2DM were included to allow for a spherical approach in the context of the study design.

Limitations of the present systematic review are inherent to the study design of the included reports, namely, the cross-sectional design of the majority of studies that does not allow for conclusions regarding causality, mechanistic insights, or precedent vascular lesions. Available studies applied various indices of AS and divergent methodological approaches to AS that are not always comparable. Due to different methodologies regarding the measurement of AS and the outcomes, statistical analysis was not feasible. Most studies were relatively old and published before the introduction of novel hypoglycemic treatments (e.g., SGLT2i, GLP-1RAs), which may favorably affect vascular function as a result of better glycemic control and concomitant weight reduction. As such, a sub-analysis according to the type of antidiabetic medication could not be performed in the present systematic review. Nevertheless, neither SGLT2i nor GLP-1RAs appear to exert a significant impact on AS based on current evidence [84–86]. Although a linear association between arterial stiffness and microvascular complications is suggested, substantial inter-study heterogeneity was observed. Due to insufficient data and limited reporting on key confounding variables such as hypertension and glycemic control, sensitivity analyses and meta-regressions could not be performed. Future research should aim to explore these relationships more thoroughly and assess the potential clinical implications of the observed association.

## Conclusion

In this systematic review, we found that increased AS correlated with microvascular impairment in divergent microvascular beds among patients with either T1DM or T2DM, and most of the time in a dose-response manner. Disease duration seems to be the leading independent risk factor for increased AS. Our systematic review suggests AS should be sought upon detection of microvascular complications in patients with T1DM or T2DM; vice versa, patients with T1DM or T2DM who present increased levels of AS should be screened for microvascular alterations. Whether the concomitant detection of AS and one or more microvascular lesions might improve cardiovascular risk stratification in patients with DM, warrants further investigation in appropriately designed future studies.

## Data Availability

Data sharing is not applicable to this article as no new data were created or analyzed in this study.

## Abbreviations

AASI: Ambulatory arterial stiffness index
Aix: Augmentation index
AS: Arterial stiffness
baPWV: Brachial-ankle pulse wave velocity
CAD: Coronary artery disease
CAVI: Carotid-ankle vascular index
cfPWV: Carotid-femoral pulse wave velocity
DM: Diabetes mellitus
HR: Hazard ratio
OCT: Optical coherence tomography
OR: Odds ratio
PAD: Peripheral arterial disease
PWV: Pulse wave velocity
T1DM: Type 1 diabetes mellitus
T2DM: Type 2 diabetes mellitus
